# Trajectories of menstrual symptoms and blood pressure in midlife: a prospective cohort study on Australian women

**DOI:** 10.1038/s41371-025-01070-0

**Published:** 2025-10-06

**Authors:** Gita D. Mishra, Chuyao Jin, Hsiu-Wen Chan, Jenny Doust, Annette Dobson

**Affiliations:** School of Public Health, The University of Queensland, Brisbane, Queensland Australia

**Keywords:** Risk factors, Hypertension

## Abstract

This study aims to investigate the associations between the trajectories of menstrual symptoms over 18 years and measured blood pressure in midlife. Data were sourced from the Australian Longitudinal Study on Women’s Health. Participants provided self-reported data on their health and wellbeing, including menstrual symptoms, through regular surveys conducted from ages 18–23 (in 1996) to ages 40–45 (in 2018). Between 2019 and 2021, a subset participated in the Menarche-to-PreMenopause study, which collected physical measurements, including blood pressure and anthropometric data. Distinct trajectories of heavy menstrual bleeding, irregular periods, and dysmenorrhea were identified among 458 women by group-based trajectory modelling. Associations between menstrual symptom trajectories and blood pressure were examined by linear regression models. After adjusting for covariates, women in the increasing heavy menstrual bleeding group had higher diastolic blood pressure (DBP) (mean difference = 3.3 mmHg, 95% CI:1.1–5.6) compared to those in the reference group. Women in chronic irregular periods group also had higher DBP (mean difference = 2.5 mmHg, 95% CI:−0.1–5.0) than those in the reference group. No differences in blood pressure were observed across the different trajectories of dysmenorrhea. In conclusion, women who experienced an increasing heavy menstrual bleeding pattern over time and chronic irregular periods during their reproductive lifespan had higher DBP in their mid-40s. This suggests that attention should be paid to the cardiovascular health of women who report menstrual symptoms.

## Introduction

Up to 91% of women of reproductive age report experiencing menstrual symptoms, including dysmenorrhea (i.e., period pain), heavy or prolonged menstrual bleeding, or irregular periods [1–3]. The prevalence of each symptom varies due to lifestyle behaviours [4, 5], genetic factors [6], reproductive history [1, 7], and gynaecological conditions [8–10]. Menstrual symptoms may affect quality of life and reproductive outcomes [5, 7, 11]. They may also be associated with long-term cardiovascular health, including blood pressure. Menstrual symptoms may be associated with long-term changes in blood pressure through processes such as endothelial dysfunction, chronic inflammation, and alterations in the renin-angiotensin-aldosterone system (RAAS). These symptoms may contribute to sustained hormonal and inflammatory effects that influence vascular function and tone [12–15].

Studies investigating the relationship between menstrual symptoms and blood pressure are often cross-sectional and typically focus on women experiencing premenstrual syndrome [14, 16, 17]. To date, there is only one study reporting the longitudinal association between menstrual symptoms and hypertension. By using time-lag models, Chung et al. [1] found a bidirectional association between heavy menstrual bleeding and chronic hypertension; and chronic hypertension was associated with the incidence of irregular periods. However, menstrual symptoms may change across the reproductive lifespan [18]. To date, no studies have examined how the experience of chronic menstrual symptoms or the emergence of symptoms at different time points throughout the reproductive lifespan may affect blood pressure.

Therefore, this study used the Australian Longitudinal Study on Women’s Health (ALSWH) dataset to investigate the association between the trajectory of menstrual symptoms – including heavy menstrual bleeding, irregular periods, and dysmenorrhea – and systolic (SBP) and diastolic blood pressure (DBP) over 18 years.

## Methods

### Study population

A total of 14247 women born in 1973–78 were recruited in the Australian Longitudinal Study on Women’s Health (ALSWH) in 1996. The participants were randomly selected from the database of the Australia’s universal health insurance scheme (Medicare). Questionnaires were sent to the women approximately every three years to collect information including their health status, lifestyle factors, and health service use. All women gave informed consent. More details of ALSWH have been reported previously [19, 20]. Between 2019 and 2021, a sub study of ALSWH – Menarche-to-PreMenopause (M-PreM) study – was conducted, with aims to elucidate the associations of reproductive factors with cardiometabolic markers, respiratory conditions, and cognitive and functional health before women reach middle age [21]. Invitations to participate in the M-PreM study were sent to all women in ALSWH 1973–78 cohort who completed Survey 8 in 2018 when they were 40–45 years old (N = 7121). Participation was impacted by the Covid epidemic and only women who lived in some major cities were able to participate in the clinical assessments. Among 4584 women who expressed interest in participating, 499 of them finished clinical assessment. Women with blood pressure measurements who had neither been diagnosed with hypertension nor used antihypertensive medications were included in the analysis (N = 458, Supplemental fig 1). Compared with women who completed Survey 8 but were not included in the analysis, those included were younger, were more likely to live in major cities, had higher education and physical activity level, were less likely to be current smokers, and had lower prevalence of endometriosis and gestational hypertension. Women included in the analysis were comparable to those not included regarding the use of oral contraceptive pill, the prevalence of other gynaecological conditions (including uterine polyps or fibroids, and polycystic ovary syndrome [PCOS]), family history of hypertension, history of gestational diabetes, and menstrual symptoms (Supplemental Table 1).

### Measurements of menstrual symptoms

We included three common menstrual symptoms in this study: heavy menstrual bleeding, irregular periods, and dysmenorrhea. In each survey, questions regarding each menstrual symptom were asked: ‘*In the last 12 months, have you had [the symptom]*’ Women were defined as having the symptom if their response was ‘often’, and as not having the symptom if their response was ‘sometimes’, ‘rarely’ or ‘never’. Data collected in Survey 1 were not included since over-reporting of menstrual symptoms was found (also known as the ‘telescoping’ effect) [18].

### Measurements of blood pressure

Study sites were established in the Translational Research Institute (Brisbane), Murdoch Children’s Research Institute (Melbourne), Royal Hospital for Women (Sydney), PARC Clinical Research (Adelaide) and Harry Perkins Institute of Medical Research (Perth). Participants were invited to attend a clinical assessment and undertook a range of biomedical measurements including blood pressure. After subjects had rested in a seated position for 5 m, three measurements of SBP and DBP were recorded with an automatic blood pressure monitor, with a 1–2 m interval between measurements. In accordance with the recommendations of the World Health Organization, the second and third measurements were averaged for analysis [22]. The brand and model of the blood pressure monitors used were shown in the Supplemental Table 2.

### Covariates

Socio-demographic covariates in Survey 8 included age (continuous), education (university degree or higher, trade/certificate/diploma, ≤12 years of schooling), and area of residence (classified as major cities and regional and remote areas based on an index of distance to the nearest urban centre [23]). Lifestyle factors reported in Survey 8 included physical activity (measured as MET-minutes per week: nil/sedentary [0–39], low [40–599], moderate [600–1199], and high [≥1200]) [24] and smoking status (never, ex-smoker, and current smoker) [25].

Gynaecological conditions included ever having a diagnosis of endometriosis, uterine polyps or fibroids, or PCOS by Survey 8. Questions about endometriosis, uterine polyps or fibroids, and PCOS were asked in Surveys 2–8, Surveys 7–8, and Surveys 4–8, respectively. Women who reported a diagnosis in any of these surveys were classified as having that condition by Survey 8. Information on the current use of oral contraceptive pills was collected in Survey 8. Pregnancy complications that may affect midlife blood pressure, including gestational hypertension and gestational diabetes, were included [26]. Family history of hypertension was assessed using the question, ‘Do you have a family history of hypertension (high blood pressure)?’ and categorised as yes, no, or don’t know.

**Fig. 1:**
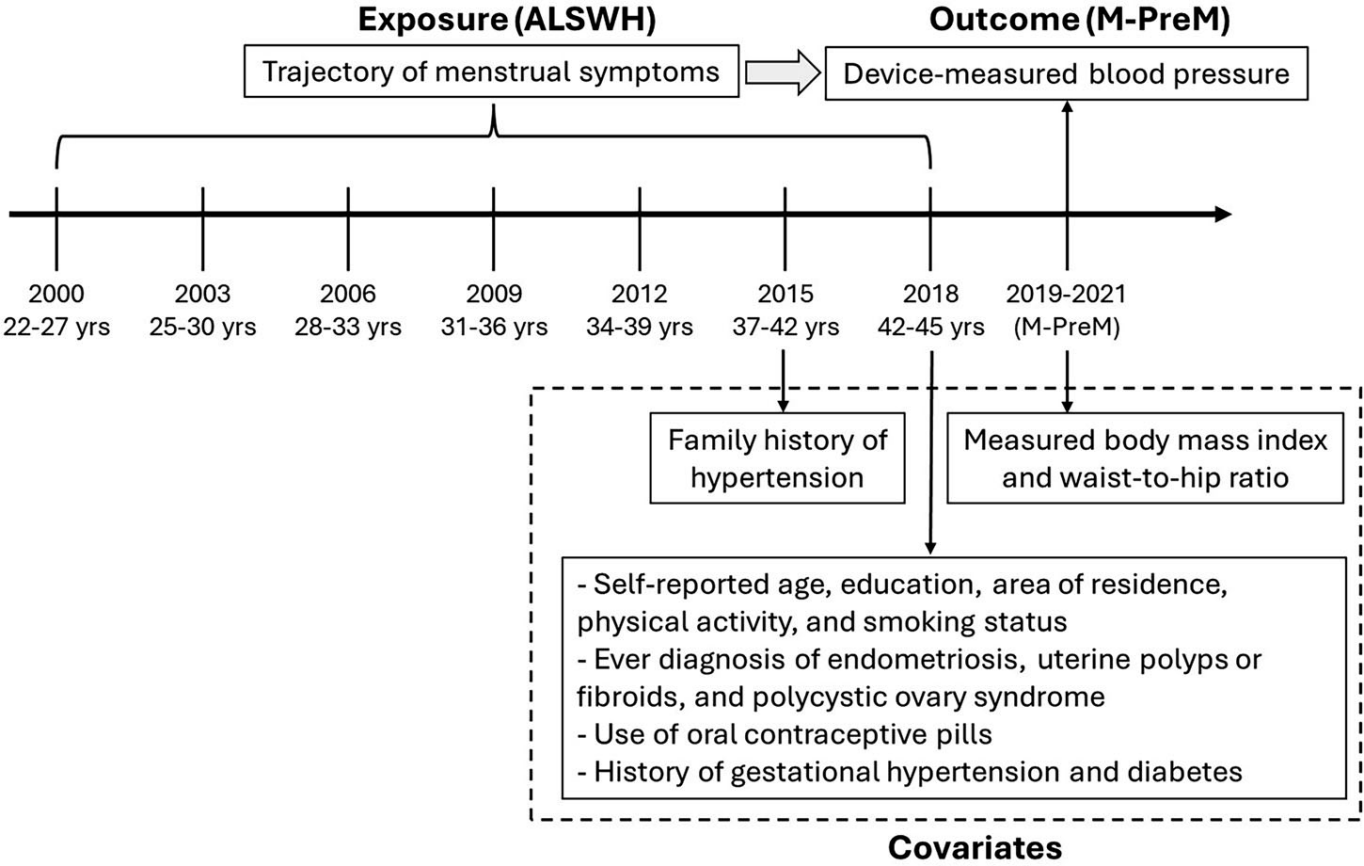
Study design diagram.

Anthropometric measurements were collected in the M-PreM study. Weight without shoes was measured once using a digital scale, accurate to 0.1 kg. Height without shoes was measured once using a stadiometer, accurate to 0.01 m. Body mass index (BMI) was calculated by dividing weight in kilograms by the square of height in meters and classified as underweight/normal weight (<25 kg/m^2^), overweight (25–<30 kg/m^2^), and obese (≥30 kg/m^2^). Waist-to-hip ratio was also included as a covariate since study has found that it might have a stronger association with cardiovascular risk than BMI [27]. The waist circumference was measured at the midpoint between the bottom of the last palpable rib and top of the iliac crest. The hip circumference was measured at the widest point of the buttocks. Waist‐to‐hip ratio was calculated by dividing the waist circumference by the hip circumference. A diagram in Fig. 1 shows when the exposure, outcome, and covariates were collected.

### Statistical analysis

Means ( ± standard deviations) and numbers (percentages) were used to describe the characteristics of the study population. Latent class growth analysis (LCGA performed using the ‘PROC TRAJ’ procedure in SAS [28]) was used to identify subgroups of participants following similar trajectories of each menstrual symptom from Survey 2 (2000) to Survey 8 (2018). The Bayesian information criterion was used to choose the best-fit model [29].

After determining the trajectories of each menstrual symptom, mean blood pressure was compared among different trajectories using the analysis of variance (ANOVA). The associations between trajectories of each menstrual symptom and differences in blood pressure were assessed by linear regression models. Covariates in the models included age, education, area of residence, physical activity, smoking status, gynaecological conditions, use of oral contraceptive pills, family history of hypertension, and history of gestational hypertension and diabetes collected in the ALSWH, and BMI and waist-to-hip ratio collected in the M-PreM study. Since blood pressure was measured using different monitors, we also included blood pressure monitors as a covariate.

Given that oral contraceptive pills can affect menstrual cycle regularity, volume, and symptoms [30], we conducted a sensitivity analysis to assess their impact in each survey. We examined the association between menstrual disorders in each survey and blood pressure, both with and without adjusting for oral contraceptive use at each time point (Survey 2 to Survey 8). All statistical analyses were performed using SAS 9.4.

## Results

The characteristics of the study population are shown in Table 1. The mean age of the subjects was 42.3 ( ± 1.5) years in Survey 8. The majority lived in major cities (93.2%) and had a university degree or higher (76.0%), and more than half (57.3%) had a measured BMI in the overweight or obese category. The proportion of women reporting often having heavy menstrual bleeding, irregular periods, and dysmenorrhea in Survey 8 were 15.1, 8.7, and 6.4%, respectively. The characteristics of the study population by the trajectories of each menstrual symptom were presented in Supplemental Table 3-5.

**Table 1:** Characteristics of participants (N = 458).

Measurements collected in Survey 8 of ALSWH	Mean ± SD or N (%)
**Age**	42.3 ± 1.5
**Area of residence**	
Major cities	425 (93.2)
Regional and remote areas	31 (6.8)
**Education**	
University degree or higher	348 (76.0)
Trade/certificate/diploma	82 (17.9)
≤12 years of schooling	28 (6.1)
**Physical activity**	
Nil/sedentary	49 (11.0)
Low	109 (24.4)
Moderate	107 (24.0)
High	181 (40.6)
**Smoking status**	
Never	320 (69.9)
Ex-smoker	111 (24.2)
Current smoker	27 (5.9)
**Current use of oral contraceptive pills**	63 (13.8)
**Ever diagnosis of gynaecological conditions by Survey 8**^α^	
Endometriosis	41 (9.0)
Uterine polyps or fibroids	27 (5.9)
Polycystic ovary syndrome	43 (9.4)
**Family history of hypertension**	
Yes	215 (49.2)
No	194 (44.4)
Not sure	28 (6.4)
**History of gestational hypertension**	27 (5.9)
**History of gestational diabetes**	31 (6.8)
**Menstrual symptoms**	
Heavy menstrual bleeding	69 (15.1)
Irregular periods	40 (8.7)
Dysmenorrhea	29 (6.4)
Measurements collected in the M-PreM study Mean ± SD or N (%)	
**Systolic blood pressure (mmHg)**	118.4 ± 12.1
**Diastolic blood pressure (mmHg)**	76.8 ± 8.6
**Body mass index**	
Underweight/normal weight (<25 kg/m^2^)	195 (42.7)
Overweight (25–<30 kg/m^2^)	138 (30.2)
Obese (≥30 kg/m^2^)	124 (27.1)
**Waist-to-hip ratio**	0.8 ± 0.1

^α^Questions about endometriosis, uterine polyps or fibroids, and polycystic ovary syndrome were asked in Surveys 2–8, Surveys 7–8, and Surveys 4–8, respectively. Women who reported a diagnosis in any of these surveys were classified as having that condition by Survey 8.

*ALSWH* Australian Longitudinal Study on Women’s Health; *M-PreM*, Menarche-to-PreMenopause.

**Fig. 2:**
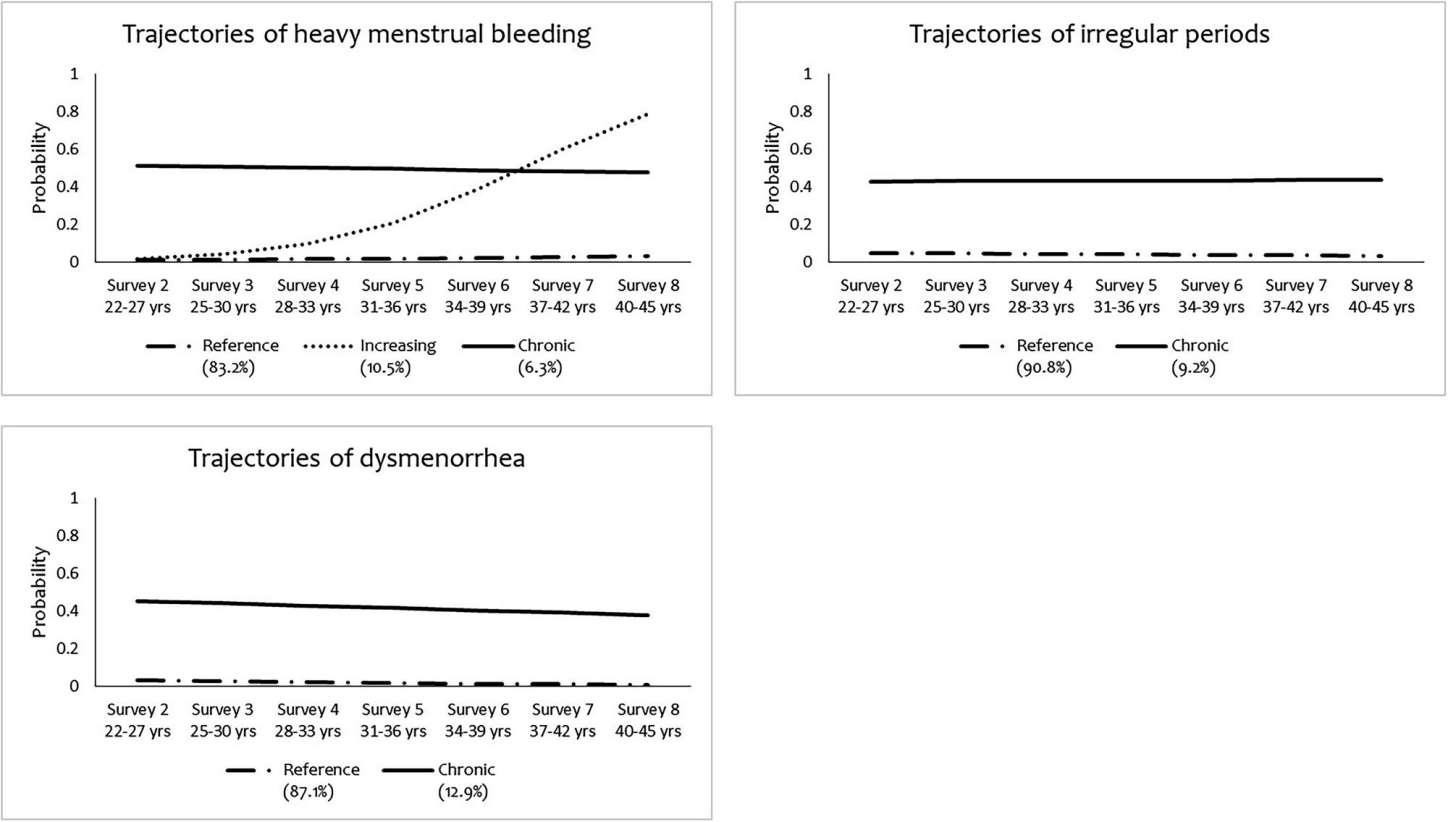
Trajectories of heavy menstrual bleeding, irregular periods, and dysmenorrhea from Survey 2 (2000) to Survey 8 (2018), N = 458.

As shown in Fig. 2, LCGA identified the following group-based trajectories (see Supplemental Table 6A-6C for model selection):3 classes for heavy menstrual bleeding (reference [83.2%], increasing [10.5%], and chronic [6.3%]), 2 classes for irregular periods (reference [90.8%] and chronic [9.2%]), and 2 classes for dysmenorrhea (reference [87.1%] and chronic [12.9%]), where ‘reference’ represented consistently low or none symptoms, ‘chronic’ represented a high probability of menstrual symptoms in each survey, and ‘increasing’ represented a symptom pattern that increased over time.

Table 2 shows the mean blood pressure by trajectories of heavy menstrual bleeding, irregular periods, and dysmenorrhea. Women in different trajectories of heavy menstrual bleeding had different DBP. For trajectories of irregular periods, women in the chronic group had higher DBP than women in the reference group (80.9 versus 76.4 mmHg), and they were comparable regarding SBP. Women in different trajectories of dysmenorrhea had similar SBP and DBP.

**Table 2:** Mean blood pressure (mmHg) by trajectories of heavy menstrual bleeding, irregular periods, and dysmenorrhea.

	Systolic blood pressure		Diastolic blood pressure	
	Mean ± SD	*P*-value	Mean ± SD	*P*-value
Trajectories of heavy menstrual bleeding				
Reference (83.2%)	117.9 ± 12.1	0.1	76.3 ± 8.6	<0.01
Increasing (10.5%)	120.8 ± 13.1		79.6 ± 8.4	
Chronic (6.3%)	121.4 ± 11.1		79.6 ± 7.6	
Trajectories of irregular periods				
Reference (90.8%)	118.2 ± 12.2	0.1	76.4 ± 8.5	<0.01
Chronic (9.2%)	121.1 ± 11.7		80.9 ± 8.2	
Trajectories of dysmenorrhea		1.0		0.4
Reference (87.1%)	118.5 ± 12.0		76.7 ± 8.6	
Chronic (12.9%)	118.4 ± 12.9		77.6 ± 8.7	

After adjusting for blood pressure monitors, socio-demographic characteristics (age, education, and area of residence), lifestyle factors (physical activity and smoking status), anthropometric measurements (BMI and waist-to-hip ratio), gynaecological conditions (endometriosis, fibroid, and PCOS), use of oral contraceptive pills, family history of hypertension, and history of gestational hypertension and diabetes, for trajectories of heavy menstrual bleeding, women in the increasing group had higher DBP than women in the reference group (mean difference:3.3 mmHg, 95% CI:1.1–5.6). For trajectories of irregular periods, women in the chronic group had higher DBP than women in the reference group, after adjusting for blood pressure monitors and socio-demographic characteristics (mean difference = 4.1 mmHg, 95% CI:1.6–6.7). The estimate was attenuated after further adjusting for lifestyle factors, anthropometric measurements, gynaecological conditions, use of oral contraceptive pills, family history of hypertension, and history of gestational hypertension and diabetes (mean difference = 2.5 mmHg, 95% CI:−0.1–5.0). The main covariates contributing to the attenuation of the estimates were BMI and PCOS. We did not find differences in blood pressure across the different trajectories of dysmenorrhea (Table 3).

**Table 3:** Mean differences in blood pressure (mmHg) between trajectory groups of heavy menstrual bleeding, irregular periods, and dysmenorrhea compared to reference groups.

	Model 1	Model 2	Model 3	Model 4
Systolic blood pressure (mmHg)				
Trajectories of heavy menstrual bleeding				
Reference (83.2%)	0 (ref)	0 (ref)	0 (ref)	0 (ref)
Increasing (10.5%)	2.8 (−0.8, 6.4)	3.0 (−0.6, 6.7)	2.8 (−0.7, 6.3)	2.7 (−0.7, 6.1)
Chronic (6.3%)	3.1 (−1.5, 7.8)	2.9 (−1.8, 7.5)	1.3 (−3.2, 5.8)	1.0 (−3.8, 5.7)
Trajectories of irregular periods				
Reference (90.8%)	0 (ref)	0 (ref)	0 (ref)	0 (ref)
Chronic (9.2%)	3.3 (−0.5, 7.1)	3.3 (−0.5, 7.1)	2.4 (−1.3, 6.0)	1.9 (−2.0, 5.7)
Trajectories of dysmenorrhea				
Reference (87.1%)	0 (ref)	0 (ref)	0 (ref)	0 (ref)
Chronic (12.9%)	−0.1 (−3.5, 3.3)	−0.1 (−3.5, 3.3)	−0.5 (−3.8, 2.7)	−0.7 (−4.0, 2.6)
Diastolic blood pressure (mmHg)				
Trajectories of heavy menstrual bleeding				
Reference (83.2%)	0 (ref)	0 (ref)	0 (ref)	0 (ref)
Increasing (10.5%)	3.1 (0.7, 5.6)	3.3 (0.8, 5.7)	3.2 (1.0, 5.5)	3.3 (1.1, 5.6)
Chronic (6.3%)	3.1 (0.0, 6.2)	2.9 (−0.3, 6.0)	1.7 (−1.2, 4.6)	1.4 (−1.8, 4.5)
Trajectories of irregular periods				
Reference (90.8%)	0 (ref)	0 (ref)	0 (ref)	0 (ref)
Chronic (9.2%)	4.2 (1.6, 6.8)	4.1 (1.6, 6.7)	3.3 (0.9, 5.7)	2.5 (−0.1, 5.0)
Trajectories of dysmenorrhea				
Reference (87.1%)	0 (ref)	0 (ref)	0 (ref)	0 (ref)
Chronic (12.9%)	0.8 (−1.5, 3.1)	0.8 (−1.5, 3.1)	0.4 (−1.7, 2.6)	0.4 (−1.7, 2.6)

Model 1 was adjusted for blood pressure monitors.

Model 2 was further adjusted for age, education, and area of residence in Survey 8.

Model 3 was further adjusted for physical activity, smoking status in Survey 8 and body mass index and waist-to-hip ratio collected in M-PreM study.

Model 4 was further adjusted for diagnosis of endometriosis, fibroid, and polycystic ovary syndrome by Survey 8, use of oral contraceptive pills in Survey 8, family history of hypertension, and history of gestational hypertension and diabetes by Survey 8.

The effects of the use of oral contraceptive pills in each survey are shown in Supplemental Table 7. The association between menstrual disorders and blood pressure remained consistent in direction and strength before and after adjustment for oral contraceptive pill use in each survey.

## Discussion

This study used longitudinal data collected over 18 years to examine the association between the trajectory of menstrual symptoms – including heavy menstrual bleeding, irregular periods, and dysmenorrhea – and measured blood pressure. The findings from this study indicate that an increasing heavy menstrual bleeding pattern over time and chronic irregular periods were associated with higher DBP. The trajectory of dysmenorrhea was not associated with blood pressure.

This study has several strengths. First, it used 18 years of longitudinal data, with surveys conducted approximately every 3 years, which enabled us to map changes in menstrual symptoms throughout the reproductive lifespan and thus build symptom trajectory profiles. Following on from this, we were able to determine how specific symptom trajectories were linked to measured blood pressure. Second, we included a wide range of potential confounders in our statistical modelling, strengthening the confidence in the results of this study.

One limitation of this study is that the definition of heavy menstrual bleeding has evolved in recent years. Previously, it was defined based on estimates of menstrual blood loss, but now it takes a more patient-centred approach. The review by the National Institute for Health and Care Excellence [31] defines heavy menstrual bleeding as “any excessive menstrual blood loss which interferes with the woman’s physical, emotional, social, and material quality of life, and which can occur alone or in combination with other symptoms”. The question used in the ALSWH survey for heavy menstrual bleeding does not use identical criteria but still reflects the symptoms women experience. Most studies validating women’s self-reported menstrual symptoms have focused on menstrual cycle length. One study, however, measured blood loss during heavy menstrual bleeding and found a link to an increased risk of hypertension [32]. This supports the association found in the current study between the self-reported heavy menstrual bleeding and higher blood pressure.

Another limitation is that the questions used to define menstrual symptoms in this study have not been validated. Women’s responses to the question “*In the last 12 months, have you had [the symptom]*” were dichotomised, with “often” as the exposed group and “sometimes,” “rarely,” and “never” grouped as unexposed, since “often” reflects frequent and persistent symptoms, while the other categories are more ambiguous. We acknowledge this approach may underestimate the true association if women reporting “sometimes” or “rarely” also have elevated risk. Additionally, these questions assessed only symptom frequency, without capturing other dimensions such as intensity and duration. It may also subject to recall bias.

In our study, we found that an increasing heavy menstrual bleeding pattern over time was associated with higher DBP. We previously reported that young women who experienced heavy menstrual bleeding had an increased risk of incident chronic hypertension, compared with those who had not experienced heavy menstrual bleeding. This relationship was also likely bidirectional, with chronic hypertension being associated with the incidence of heavy menstrual bleeding [1]. The current study confirms previous reports of an association between heavy menstrual bleeding and high blood pressure. It also demonstrates that the trajectory of the symptom matters; women who experienced an increasing heavy menstrual bleeding pattern over time had higher DBP. Heavy menstrual bleeding is more likely to occur in older women as they approach menopause due to estrogen fluctuations, and increased risk of developing polyps or endometrial hyperplasia [33]. Heavy menstrual bleeding has been reported to double in prevalence among women surveyed between the ages of 22 and 42 over a 15-year period, suggesting that a high proportion of these women are likely to have an increasing heavy menstrual bleeding pattern over time [4] and thus, are at an increased risk of high blood pressure.

A previous study reported an association between chronic hypertension and incident irregular periods but the association did not appear to be bidirectional [1]. The current study further characterises the complexity of this association by demonstrating that women with chronic irregular periods from their 20–40 s had higher DBP. However, this association was attenuated after controlling for covariates, particularly when gynaecological conditions were introduced into the models. This suggests that the association may be explained by gynaecological conditions such as PCOS, which is characterised by irregular periods and linked to high blood pressure [34].

The absence of an association between dysmenorrhea and blood pressure is not surprising, given that previous studies using the same cohort have not identified such a link [1]. However, a Japanese cohort study found that painful periods around the age of 20, rather than just prior to pregnancy, were associated with an increased risk of hypertensive disorder of pregnancy [35], highlighting that the long term associations between painful periods on blood pressure should not be discounted.

Both trajectories of heavy menstrual bleeding and irregular periods were associated with higher DBP. In the Framingham Heart Study, DBP was found to be the strongest predictor of coronary heart disease risk, rather than SBP or pulse pressure, among participants under 50 years of age [36]. Similarly, another study that included 12 randomly recruited population cohorts found that, below age 50, DBP and isolated diastolic hypertension were the main drivers of coronary complications [37]. Given these findings, further replication of the association between menstrual symptoms and DBP in other cohorts and age groups is needed to determine whether monitoring DBP in women with an increasing heavy menstrual bleeding pattern over time and chronic irregular periods—particularly before age 50—is beneficial.

In our study, women in the chronic irregular periods group had 2.5 mm Hg higher DBP than those in the reference group. Even modest increases in DBP may elevate cardiovascular risk. According to a large meta-analysis of over one million adults [38], a 10 mm Hg lower usual DBP at age 40 was associated with hazard ratios of 0.35 for stroke death, 0.47 for ischaemic heart disease death, and 0.43 for death from other vascular causes between ages 40 and 49. These findings underscore the importance of managing even small elevations in DBP to reduce cardiovascular risk in middle-aged adults.

In conclusion, our results showed that women with an increasing heavy menstrual bleeding pattern over time or chronic irregular periods had higher DBP in midlife. Given that elevated blood pressure is a major risk factor for cardiovascular disease, the associations we observed require replication in diverse cohorts and age groups to determine whether routine monitoring of menstrual symptoms and blood pressure is beneficial for promoting cardiovascular health in midlife.

## Summary

### What is known about topic?

- Menstrual symptoms have been linked to blood pressure.
- No previous studies have investigated how chronic menstrual symptoms or the timing of symptom onset throughout a woman’s reproductive lifespan may affect blood pressure.

### What this study adds?

- Our findings highlight that the timing of symptom onset matters—women who experienced an increasing heavy menstrual bleeding pattern over time and chronic irregular periods were more likely to have higher blood pressure in middle age.
- Our study suggests a potential link between menstrual symptoms and high blood pressure. Replication in diverse populations is needed to assess whether monitoring these symptoms could support early detection or management of high blood pressure.

## Supplementary information

Supplemental materials

## Data Availability

Data are available upon reasonable request. Access to the M-PreM dataset requires approval from the Australian Longitudinal Study on Women’s Health (ALSWH) Data Access Committee. More information can be found at the ALSWH website: https://alswh.org.au/for-data-users/.

## Code availability

The code used for data processing and analysis in this study is available from the corresponding author upon reasonable request.
