# Supplementary material for: Trajectories of menstrual symptoms and blood pressure in midlife: a prospective cohort study on Australian women
Source: J Hum Hypertens. 2025 Oct 6;39(12):874–80. doi: 10.1038/s41371-025-01070-0 (PMC12685742; doi:10.1038/s41371-025-01070-0)
Supplement: Supplementary file 1 — Supplemental materials [file 41371_2025_1070_MOESM1_ESM.docx]

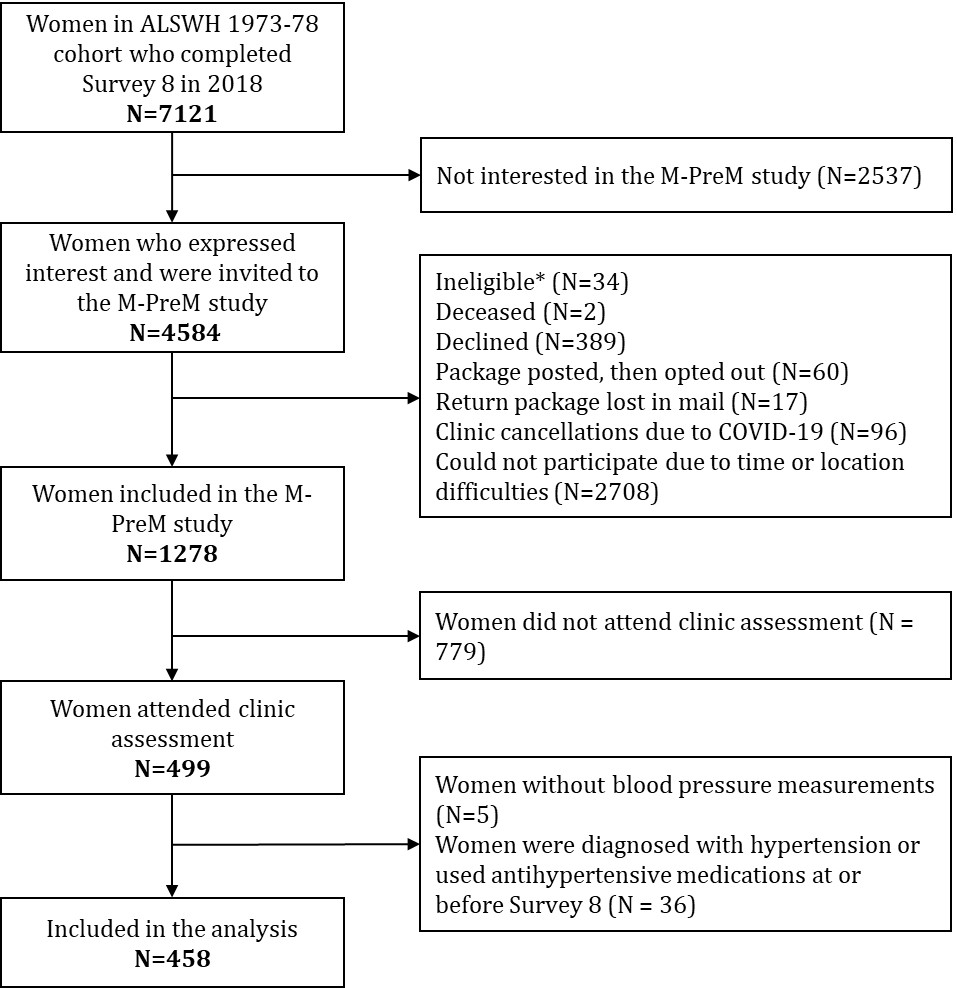


**Supplemental Figure 1.** Flow chart of the selection of participants

* Women who were pregnant or were being treated for a reproductive cancer at the time of invitation were excluded.

**Supplemental Table 1.** Comparison of characteristics in Survey 8 (2018) between women included in the analysis and women not included

|  | Women included in the analysis (n=458) | Women not included in the analysis (n=6663) | *P*-value |
| --- | --- | --- | --- |
| Age, mean±SD | 42.3 ± 1.5 | 42.4 ± 1.5 | <0.01 |
| Area of residence, N (%) |  |  | <0.01 |
| Major cities | 425 (93.2) | 3599 (55.6) |  |
| Reginal and remote areas | 31 (6.8) | 2875 (44.4) |  |
| Education, N (%) |  |  | <0.01 |
| University degree or higher | 348 (76.0) | 3546 (56.1) |  |
| Trade/certificate/diploma | 82 (17.9) | 1825 (28.9) |  |
| ≤12 years of schooling | 28 (6.1) | 947 (15.0) |  |
| Physical activity, N (%) |  |  | <0.01 |
| Nil/sedentary | 49 (11.0) | 773 (13.3) |  |
| Low | 109 (24.4) | 1797 (30.8) |  |
| Moderate | 107 (24.0) | 1305 (22.4) |  |
| High | 181 (40.6) | 1956 (33.5) |  |
| Smoking status, N (%) |  |  | <0.01 |
| Never | 320 (69.9) | 3934 (61.1) |  |
| Ex-smoker | 111 (24.2) | 1836 (28.5) |  |
| Current smoker | 27 (5.9) | 669 (10.4) |  |
| Current use of oral contraceptive pills, N (%) | 63 (13.8) | 840 (13.2) | 0.7 |
| Ever diagnosis of gynaecological conditions by Survey 8^α^ |  |  |  |
| Endometriosis, N (%) | 41 (9.0) | 842 (12.6) | 0.02 |
| Uterine polyps or fibroids, N (%) | 27 (5.9) | 321 (4.8) | 0.3 |
| Polycystic ovary syndrome, N (%) | 43 (9.4) | 626 (9.4) | 1.0 |
| **Family history of hypertension**, N (%) |  |  | 0.4 |
| Yes | 215 (49.2) | 2884 (52.6) |  |
| No | 194 (44.4) | 2252 (41.1) |  |
| Not sure | 28 (6.4) | 344 (6.3) |  |
| **History of gestational hypertension**, N (%) | 27 (5.9) | 756 (11.4) | <0.01 |
| **History of gestational diabetes**, N (%) | 31 (6.8) | 562 (8.4) | 0.2 |
| Menstrual symptoms |  |  |  |
| Heavy menstrual bleeding, N (%) | 69 (15.1) | 799 (12.4) | 0.09 |
| Irregular periods, N (%) | 40 (8.7) | 436 (6.8) | 0.1 |
| Dysmenorrhea, N (%) | 29 (6.4) | 484 (7.5) | 0.4 |

^α^The questions regarding endometriosis, uterine polyps or fibroids, and polycystic ovary syndrome were asked between Surveys 2-8, Surveys 7-8, and Surveys 4-8, respectively. Women reported having diagnosis in any survey were classified as having that condition by Survey 8.

**Supplemental Table 2.** The brand and model of the blood pressure monitors used in the Menarche-to-PreMenopause (M-PreM) study

| Study location | Brand | Model |
| --- | --- | --- |
| Brisbane, Australia | Welch Allyn | Connex® Vital Signs Machine 6000 Series |
| Melbourne, Australia | Welch Allyn | Connex® ProBP™ 3400 Digital Blood Pressure Device |
| Adelaide, Australia | Phillips | Intellivue MP30 Patient Monitor |
| Perth, Australia | Omron | Automatic Blood Pressure Monitor HEM-907 |
| Sydney, Australia | Omron | Automatic Blood Pressure Monitor HEM-7121 |

**Supplemental Table 3.** Comparison of characteristics in Survey 8 (2018) between women in different trajectories of heavy menstrual bleeding

|  | Trajectory of heavy menstrual bleeding | | | *P*-value |
| --- | --- | --- | --- | --- |
|  | Reference  (N = 381, 83.2%) | Increasing  (N = 48, 10.5%) | Chronic  (N = 29, 6.3%) |  |
| Age, mean±SD | 42.2 ± 1.5 | 42.4 ± 1.5 | 42.2 ± 1.8 | 0.8 |
| Area of residence, N (%) |  |  |  | 0.7 |
| Major cities | 351 (92.6) | 46 (95.8) | 28 (96.6) |  |
| Reginal and remote areas | 28 (7.4) | 2 (4.2) | 1 (3.5) |  |
| Education, N (%) |  |  |  | 0.1 |
| University degree or higher | 290 (76.1) | 38 (79.2) | 20 (69.0) |  |
| Trade/certificate/diploma | 68 (17.9) | 5 (10.4) | 9 (31.0) |  |
| ≤12 years of schooling | 23 (6.0) | 5 (10.4) | 0 (0.0) |  |
| Physical activity, N (%) |  |  |  | 0.5 |
| Nil/sedentary | 39 (10.5) | 5 (10.6) | 5 (17.9) |  |
| Low | 91 (24.5) | 12 (25.5) | 6 (21.4) |  |
| Moderate | 87 (23.5) | 10 (21.3) | 10 (35.7) |  |
| High | 154 (41.5) | 20 (42.6) | 7 (25.0) |  |
| Smoking status, N (%) |  |  |  | 0.01 |
| Never | 275 (72.2) | 31 (64.6) | 14 (48.3) |  |
| Ex-smoker | 89 (23.4) | 11 (22.9) | 11 (37.9) |  |
| Current smoker | 17 (4.5) | 6 (12.5) | 4 (13.8) |  |
| Current use of oral contraceptive pills, N (%) | 54 (14.2) | 6 (12.8) | 3 (10.7) | 0.9 |
| Ever diagnosis of gynaecological conditions by Survey 8^α^ |  |  |  |  |
| Endometriosis, N (%) | 25 (6.6) | 7 (14.6) | 9 (31.0) | <0.01 |
| Uterine polyps or fibroids, N (%) | 15 (3.9) | 3 (6.3) | 9 (31.0) | <0.01 |
| Polycystic ovary syndrome, N (%) | 33 (8.7) | 4 (8.3) | 6 (20.7) | 0.1 |
| **Family history of hypertension**, N (%) |  |  |  | 0.04 |
| Yes | 174 (47.5) | 22 (46.8) | 19 (79.2) |  |
| No | 169 (46.2) | 21 (44.7) | 4 (16.7) |  |
| Not sure | 23 (6.3) | 4 (8.5) | 1 (4.2) |  |
| **History of gestational hypertension**, N (%) | 20 (5.3) | 4 (8.3) | 3 (10.3) | 0.3 |
| **History of gestational diabetes**, N (%) | 26 (6.8) | 3 (6.3) | 2 (6.9) | 1.0 |
| Body mass index, N (%)^β^ |  |  |  | 0.1 |
| Underweight/normal weight (<25kg/m^2^) | 170 (44.7) | 19 (39.6) | 6 (20.7) |  |
| Overweight (25 to <30kg/m^2^) | 109 (28.7) | 18 (37.5) | 11 (37.9) |  |
| Obese (≥30kg/m^2^) | 101 (26.6) | 11 (22.9) | 12 (41.4) |  |
| Waist-to-hip ratio, mean±SD ^β^ | 0.8 ± 0.1 | 0.8 ± 0.1 | 0.9 ± 0.1 | 0.04 |

^α^Questions about endometriosis, uterine polyps or fibroids, and polycystic ovary syndrome were asked in Surveys 2-8, Surveys 7-8, and Surveys 4-8, respectively. Women who reported a diagnosis in any of these surveys were classified as having that condition by Survey 8.

^β^Measurements were collected in the Menarche-to-PreMenopause (M-PreM) study.

**Supplemental Table 4.** Comparison of characteristics in Survey 8 (2018) between women in different trajectories of irregular periods

|  | Trajectory of irregular periods | | *P*-value |
| --- | --- | --- | --- |
|  | Reference  (N = 416, 90.8%) | Chronic  (N = 42, 9.2%) |  |
| Age, mean±SD | 42.3 ± 1.5 | 41.9 ± 1.6 | 0.1 |
| Area of residence, N (%) |  |  |  |
| Major cities | 386 (93.0) | 39 (95.1) | 1.0 |
| Reginal and remote areas | 29 (7.0) | 2 (4.9) |  |
| Education, N (%) |  |  | 0.5 |
| University degree or higher | 319 (76.7) | 29 (69.1) |  |
| Trade/certificate/diploma | 73 (17.6) | 9 (21.4) |  |
| ≤12 years of schooling | 24 (5.8) | 4 (9.5) |  |
| Physical activity, N (%) |  |  | 0.4 |
| Nil/sedentary | 42 (10.4) | 7 (16.7) |  |
| Low | 96 (23.8) | 13 (31.0) |  |
| Moderate | 99 (24.5) | 8 (19.1) |  |
| High | 167 (41.3) | 14 (33.3) |  |
| Smoking status, N (%) |  |  | 0.7 |
| Never | 289 (69.5) | 31 (73.8) |  |
| Ex-smoker | 103 (24.8) | 8 (19.1) |  |
| Current smoker | 24 (5.8) | 3 (7.1) |  |
| Current use of oral contraceptive pills, N (%) | 58 (14.0) | 5 (11.9) | 0.7 |
| Ever diagnosis of gynaecological conditions by Survey 8^α^ |  |  |  |
| Endometriosis, N (%) | 37 (8.9) | 4 (9.5) | 0.9 |
| Uterine polyps or fibroids, N (%) | 21 (5.1) | 6 (14.3) | 0.03 |
| Polycystic ovary syndrome, N (%) | 26 (6.3) | 17 (40.5) | <0.01 |
| **Family history of hypertension**, N (%) |  |  | 0.7 |
| Yes | 193 (48.6) | 22 (55.0) |  |
| No | 178 (44.8) | 16 (40.0) |  |
| Not sure | 26 (6.6) | 2 (5.0) |  |
| **History of gestational hypertension**, N (%) | 23 (5.5) | 4 (9.5) | 0.3 |
| **History of gestational diabetes**, N (%) | 23 (5.5) | 8 (19.1) | <0.01 |
| Body mass index, N (%)^β^ |  |  | 0.2 |
| Underweight/normal weight (<25kg/m^2^) | 183 (44.1) | 12 (28.6) |  |
| Overweight (25 to <30kg/m^2^) | 122 (29.4) | 16 (38.1) |  |
| Obese (≥30kg/m^2^) | 110 (26.5) | 14 (33.3) |  |
| Waist-to-hip ratio, mean±SD^β^ | 0.8 ± 0.1 | 0.8 ± 0.1 | 0.7 |

^α^Questions about endometriosis, uterine polyps or fibroids, and polycystic ovary syndrome were asked in Surveys 2-8, Surveys 7-8, and Surveys 4-8, respectively. Women who reported a diagnosis in any of these surveys were classified as having that condition by Survey 8.

^β^Measurements were collected in the Menarche-to-PreMenopause (M-PreM) study.

**Supplemental Table 5.** Comparison of characteristics in Survey 8 (2018) between women in different trajectories of dysmenorrhea

|  | Trajectory of dysmenorrhea | | *P*-value |
| --- | --- | --- | --- |
|  | Reference  (N = 399, 87.1%) | Chronic  (N = 59, 12.9%) |  |
| Age, mean±SD | 42.2 ± 1.5 | 42.6 ± 1.6 | 0.1 |
| Area of residence, N (%) |  |  | 0.4 |
| Major cities | 368 (92.7) | 57 (96.6) |  |
| Reginal and remote areas | 29 (7.3) | 2 (3.4) |  |
| Education, N (%) |  |  | 0.2 |
| University degree or higher | 308 (77.2) | 40 (67.8) |  |
| Trade/certificate/diploma | 67 (16.8) | 15 (25.4) |  |
| ≤12 years of schooling | 24 (6.0) | 4 (6.8) |  |
| Physical activity, N (%) |  |  | 0.5 |
| Nil/sedentary | 40 (10.3) | 9 (15.8) |  |
| Low | 93 (23.9) | 16 (28.1) |  |
| Moderate | 95 (24.4) | 12 (21.1) |  |
| High | 161 (41.4) | 20 (35.1) |  |
| Smoking status, N (%) |  |  | 0.6 |
| Never | 281 (70.4) | 39 (66.1) |  |
| Ex-smoker | 96 (24.1) | 15 (25.4) |  |
| Current smoker | 22 (5.5) | 5 (8.5) |  |
| Current use of oral contraceptive pills, N (%) | 56 (14.0) | 7 (12.3) | 0.7 |
| Ever diagnosis of gynaecological conditions by Survey 8^α^ |  |  |  |
| Endometriosis, N (%) | 25 (6.3) | 16 (27.1) | <0.01 |
| Uterine polyps or fibroids, N (%) | 20 (5.0) | 7 (11.9) | 0.1 |
| Polycystic ovary syndrome, N (%) | 33 (8.3) | 10 (17.0) | 0.03 |
| **Family history of hypertension**, N (%) |  |  | 0.05 |
| Yes | 186 (48.6) | 29 (53.7) |  |
| No | 176 (46.0) | 18 (33.3) |  |
| Not sure | 21 (5.5) | 7 (13.0) |  |
| **History of gestational hypertension**, N (%) | 21 (5.3) | 6 (10.2) | 0.1 |
| **History of gestational diabetes**, N (%) | 28 (7.0) | 3 (5.1) | 0.8 |
| Body mass index, N (%)^β^ |  |  | 0.4 |
| Underweight/normal weight (<25kg/m^2^) | 174 (43.7) | 21 (35.6) |  |
| Overweight (25 to <30kg/m^2^) | 120 (30.2) | 18 (30.5) |  |
| Obese (≥30kg/m^2^) | 104 (26.1) | 20 (33.9) |  |
| Waist-to-hip ratio, mean±SD^β^ | 0.8 ± 0.1 | 0.8 ± 0.1 | 0.1 |

^α^Questions about endometriosis, uterine polyps or fibroids, and polycystic ovary syndrome were asked in Surveys 2-8, Surveys 7-8, and Surveys 4-8, respectively. Women who reported a diagnosis in any of these surveys were classified as having that condition by Survey 8.

^β^Measurements were collected in the Menarche-to-PreMenopause (M-PreM) study.

**Supplemental Table 6A.** Model fit statistics for latent class growth models to detect trajectories of heavy menstrual bleeding from Survey 2 (2000) to Survey 8 (2018), N=458

| Number of classes | BIC | Class 1,  N (%) | Class 2,  N (%) | Class 3,  N (%) | Class 4,  N (%) |
| --- | --- | --- | --- | --- | --- |
| 1 | -899.11 | 100 | NA | NA | NA |
| 2 | -791.85 | 68.1 | 31.9 | NA | NA |
| **3** | **-780.93** | **80.3** | **12.4** | **7.4** | **NA** |
| 4 | -786.78 | 57.5 | 29.9 | 9.1 | 3.5 |

BIC indicated the 3-class model was the optimal representation of the data.

BIC = Bayesian Information Criterion.

**Supplemental Table 6B.** Model fit statistics for latent class growth models to detect trajectories of irregular periods from Survey 2 (2000) to Survey 8 (2018), N=458

| Number of classes | BIC | Class 1,  N (%) | Class 2,  N (%) | Class 3,  N (%) | Class 4,  N (%) |
| --- | --- | --- | --- | --- | --- |
| 1 | -841.44 | 100 | NA | NA | NA |
| **2** | **-782.28** | **89.6** | **10.4** | **NA** | **NA** |
| 3 | -785.02 | 18.9 | 73.8 | 7.3 | NA |
| 4 | -794.21 | 73.8 | 7.3 | 10.2 | 8.7 |

BIC indicated the 2-class model was the optimal representation of the data.

BIC = Bayesian Information Criterion.

**Supplemental Table 6C.** Model fit statistics for latent class growth models to detect trajectories of dysmenorrhea from Survey 2 (2000) to Survey 8 (2018), N=458

| Number of classes | BIC | Class 1,  N (%) | Class 2,  N (%) | Class 3,  N (%) | Class 4,  N (%) |
| --- | --- | --- | --- | --- | --- |
| 1 | -785.79 | 100 | NA | NA | NA |
| **2** | **-669.05** | **85.7** | **14.3** | **NA** | **NA** |
| 3 | -673.96 | 76.2 | 16.2 | 7.7 | NA |
| 4 | -677.60 | 22.9 | 68.2 | 0.8 | 8.1 |

BIC indicated the 2-class model was the optimal representation of the data.

BIC = Bayesian Information Criterion.

**Supplemental Table 7.** Association between menstrual disorders in each survey and blood pressure, before and after adjusting for oral contraceptive pill use in the respective survey

| Menstrual disorders  (often vs sometimes/rarely/never) | Systolic blood pressure | |  | Diastolic blood pressure | |
| --- | --- | --- | --- | --- | --- |
|  | Crude model | Adjusted for oral contraceptive pills |  | Crude model | Adjusted for oral contraceptive pills |
| Heavy menstrual bleeding |  |  |  |  |  |
| Survey 2 | 4.7 (-0.5, 10.0) | 4.7 (-0.6, 10.1) |  | 3.2 (-0.5, 6.9) | 2.6 (-1.1, 6.4) |
| Survey 3 | 3.6 (-2.9, 10.1) | 3.7 (-2.8, 10.2) |  | 2.2 (-2.4, 6.8) | 2.1 (-2.5, 6.8) |
| Survey 4 | 0.3 (-4.6, 5.2) | 0.0 (-5.0, 5.0) |  | 2.9 (-0.6, 6.4) | 2.7 (-0.8, 6.2) |
| Survey 5 | 3.5 (-0.7, 7.7) | 3.7 (-0.5, 7.9) |  | 3.4 (0.4, 6.4) | 3.6 (0.6, 6.5) |
| Survey 6 | 1.2 (-2.2, 4.7) | 1.5 (-1.9, 5.0) |  | 2.3 (-0.1, 4.7) | 2.5 (0.1, 4.9) |
| Survey 7 | 1.3 (-2.3, 4.8) | 1.8 (-1.7, 5.3) |  | 1.1 (-1.4, 3.5) | 1.5 (-1.0, 3.9) |
| Survey 8 | 3.3 (0.2, 6.5) | 3.5 (0.4, 6.6) |  | 4.0 (1.9, 6.2) | 4.1 (1.9, 6.3) |
| Irregular periods |  |  |  |  |  |
| Survey 2 | 1.0 (-3.1, 5.2) | 0.9 (-3.4, 5.1) |  | 0.5 (-2.4, 3.5) | -0.1 (-3.1, 2.9) |
| Survey 3 | -1.3 (-5.7, 3.2) | -1.2 (-5.7, 3.3) |  | 0.8 (-2.3, 3.9) | 0.6 (-2.6, 3.8) |
| Survey 4 | -0.6 (-5.1, 3.9) | -0.8 (-5.4, 3.7) |  | 4.0 (0.8, 7.2) | 3.9 (0.7, 7.1) |
| Survey 5 | 6.7 (2.5, 10.9) | 6.8 (2.6, 10.9) |  | 4.2 (1.2, 7.2) | 4.3 (1.3, 7.3) |
| Survey 6 | 3.1 (-1.2, 7.4) | 3.2 (-1.1, 7.5) |  | 4.4 (1.5, 7.4) | 4.5 (1.5, 7.5) |
| Survey 7 | 1.8 (-2.7, 6.3) | 2.1 (-2.3, 6.6) |  | 2.0 (-1.1, 5.2) | 2.3 (-0.8, 5.4) |
| Survey 8 | 0.0 (-3.9, 3.9) | 0.2 (-3.7, 4.1) |  | 2.0 (-0.7, 4.8) | 2.1 (-0.7, 4.9) |
| Dysmenorrhea |  |  |  |  |  |
| Survey 2 | -0.6 (-4.5, 3.4) | -0.8 (-4.8, 3.3) |  | 0.1 (-2.8, 2.9) | -0.5 (-3.4, 2.4) |
| Survey 3 | -1.4 (-6.3, 3.6) | -1.4 (-6.3, 3.6) |  | -1.9 (-5.4, 1.6) | -2.0 (-5.5, 1.5) |
| Survey 4 | -1.4 (-6.2, 3.5) | -1.5 (-6.3, 3.3) |  | -1.5 (-4.9, 1.9) | -1.6 (-5.0, 1.8) |
| Survey 5 | -0.1 (-4.6, 4.4) | 0.0 (-4.5, 4.5) |  | -0.6 (-3.8, 2.6) | -0.4 (-3.6, 2.8) |
| Survey 6 | 3.3 (-1.0, 7.6) | 3.4 (-0.9, 7.6) |  | 1.9 (-1.1, 4.9) | 1.9 (-1.1, 4.9) |
| Survey 7 | 3.2 (-1.4, 7.8) | 3.9 (-0.7, 8.4) |  | 2.3 (-0.9, 5.5) | 2.8 (-0.4, 6.0) |
| Survey 8 | 3.3 (-1.3, 7.9) | 3.3 (-1.3, 7.9) |  | 2.6 (-0.7, 5.9) | 2.6 (-0.7, 5.8) |
